# Regulation of Chemerin and CMKLR1 Expression by Nutritional Status, Postnatal Development, and Gender

**DOI:** 10.3390/ijms19102905

**Published:** 2018-09-25

**Authors:** Estrella Sanchez-Rebordelo, Juan Cunarro, Sonia Perez-Sieira, Luisa María Seoane, Carlos Diéguez, Ruben Nogueiras, Sulay Tovar

**Affiliations:** 1 Departamento de Fisioloxía, Centro de Investigación en Medicina Molecular (CIMUS), Universidade de Santiago de Compostela-Instituto de Investigación Sanitaria de Santiago de Compostela (IDIS), 15782 Santiago de Compostela, Spain; estrellasanchezrebordelo@gmail.com (E.S.-R.); juan.cg.1992@gmail.com (J.C.); sonia.perez.sieira@gmail.com (S.P.-S.); carlos.dieguez@usc.es (C.D.); ruben.nogueiras@usc.es (R.N.); 2 CIBER Fisiopatología de la Obesidad y Nutrición (CIBERobn), 15706 Santiago de Compostela, Spain; luisamaria.seoane@usc.es; 3 Grupo Fisiopatología Endocrina, Instituto de Investigación Sanitaria de Santiago de Compostela, Complexo Hospitalario Universitario de Santiago (CHUS/SERGAS), 15706 Santiago de Compostela, Spain

**Keywords:** white adipose tissue, adipokine, leptin, hormonal status

## Abstract

Chemerin (also known as tazarotene-induced gene 2 and retinoic acid receptor responder 2) has been identified as an adipokine that exerts effects on many biological processes, including adipogenesis, angiogenesis, inflammation, immune responses, and food intake. This variety of effects has led to its implication in obesity and co-morbidities including diabetes and a risk of cardiovascular disease. The biological effects are mostly mediated by a so-called G protein-coupled receptor, chemokine-like receptor 1 (CMKLR1). Given the association of chemerin with obesity and related diseases, we decided to study in detail the regulation of chemerin and CMKLR1 expression in white adipose tissue (WAT). Specifically, we focused on their expression levels in physiological and pathophysiological settings involved in energy balance: e.g., fasting, postnatal development, and gender. We used Sprague Dawley rats with different nutritional statuses, levels of hormonal deficiency, and states of development as well as ob/ob (leptin-deficient) mice. We analysed the protein expression of both the ligand and receptor (chemerin and CMKLR1) in gonadal WAT by western blotting. We found that chemerin and CMKLR1 protein levels were regulated in WAT by different conditions associated with metabolic changes such as nutritional status, sex steroids, pregnancy, and food composition. Our data indicate that regulation of the expression of this new adipokine and its receptor by nutritional status and gonadal hormones may be a part of the adaptive mechanisms related to altered fat mass and its metabolic complications.

## 1. Introduction

Obesity is a worldwide health problem, and in the last 50 years, the global rate of obesity increased dramatically not only among adults but also among children where obesity has reached an incidence of up to 17% in the USA [1,2]. Obesity is characterised by an excess of adipose tissue and lipid storage in an ectopic manner. Accordingly, the incidence of type 2 diabetes—that developed as a consequence of insulin resistance, which leads to hyperglycaemia—has increased in parallel and contributes to increased morbidity and mortality [3].

Data gleaned over the last years unmasked adipose tissue as an active endocrine organ that secretes different molecules into the circulation known as adipokines [4,5,6]. They have many functions such as adipose-tissue development, an influence on the regulation of glucose and lipid metabolism, inflammation, and immune function in addition to other, tissue-specific effects exerted via an endocrine mechanism in such organs as the brain, liver, and skeletal muscle [7]. For this reason, the knowledge about the mechanisms underlying gene expression of the different adipokines and their receptors is essential to expand our understanding of adiposity and metabolic homeostasis. One of these adipokines, which aroused stronger interest in recent years, is chemerin.

Chemerin has been identified as tazarotene-induced gene 2 (TIG2) and retinoic acid receptor responder (RARRES2) and is described as a chemoattractant factor for the leukocyte population. Chemerin is synthesised in an inactive protein form, thereafter undergoing cleavage and processing at either serum or tissue levels. This process (poorly characterised) leads to the production of different chemerin isoforms that may possess different bioactivities and functions [8,9,10].

Chemerin has been identified as the ligand of the orphan G protein-coupled receptor chemokine-like receptor (CMKLR1), which shares a similarity with a chemokine receptor and is thought to be the receptor through which chemerin exerts most of its biological effects related to inflammation [11]. Although more receptors have been identified such as G protein-coupled receptor GPR1, whose expression is predominant in the central nervous system and, thus, is unlikely to mediate many of the direct effects exerted directly by the ligand in peripheral tissues. Another postulated receptor is chemokine (C-C motif) receptor-like 2 (CCRL2) whose relevance is not as clear because it has a much lower affinity for chemerin [12,13,14].

Chemerin is expressed most abundantly in the liver and in white adipose tissue (WAT), moderately in lungs and brown adipose tissue, and weakly in the heart, ovaries, and kidneys [15,16]. CMKLR1 is expressed principally in immature dendritic cells and macrophages [9] and at lower levels in the lungs, brain, heart, and placenta [16]. WAT is a unique tissue in which researchers have found high levels of chemerin and CMKLR1. Goralski [16] has proposed a hypothesis that this tissue is a source of chemerin and one of the main targets for its biological actions. Thus, it has been demonstrated that the loss of chemerin and CMKLR1 abrogates adipocyte differentiation and modifies the expression of genes critical for glucose and lipid metabolism [11,16].

Chemerin acting through CMKLR1 has both anti- and pro-inflammatory properties. The pro-inflammatory properties lead to chemoattractant effect for leukocytes to sites of inflammation [9,17]. In addition, chemerin promotes leakage of macrophages into the extravascular compartment and leakage of adhesion molecules facilitating adhesion of macrophages to the endothelium [18]. In humans, the pro-inflammatory role arose from the data showing that serum levels of chemerin positively correlate with interleukin 6 (IL-6), C-reactive protein, and tumor necrosis factor α (TNF-α) levels [19,20]. Experimental evidence has also been reported in support of the anti-inflammatory properties in relation to the resolution of inflammation [21]. Nonetheless, more relevant results to this effect have been described in a mouse model of lipopolysaccharide (LPS)-induced lung inflammation, where administration of recombinant chemerin decreases lung tissue inflammation and alveolar infiltration by neutrophils compared to vehicle-treated mice. This effect is quite specific because CMKLR1 knockout (KO) mice, in contrast to wild-type animals, do not manifest any response to the beneficial effects of chemerin and show elevated LPS-induced neutrophil accumulation [22]. Taken together, these observations indicate that the chemerin–CMKLR1 signalling system plays a key role as a mediator of LPS-induced inflammation.

Further interest in this adipokine comes from the impaired glucose-stimulated insulin secretion observed in chemerin- and CMKLR1-KO mice [23,24]. Besides, in humans, the expression of this adipokine is influenced by metabolic status. Chemerin levels increase during the development of impaired glucose tolerance and type 2 diabetes [25] and correlate with the body–mass index and waist-to-hip ratio [15,26]. In addition, in obese patients, chemerin appears to have different isoform profiles in plasma and in WAT [27].

Various studies have been conducted to try to understand the regulation of chemerin expression in different tissues by metabolic and physiological parameters or relevant diseases. Furthermore, there are many reports regarding chemerin levels in different pathophysiological settings, although, it should be noted that available immunoassays have an important limitation, namely, they are unable to differentiate between the active and inactive forms of this protein Nonetheless, until now, there has not been any clear evidence about the regulation of chemerin and CMKLR1 expression in one of the most important sources of chemerin and CMKLR1: WAT. This question is quite important because chemerin undergoes tissue-specific post-translational processing. Therefore, the aim of this study was to investigate the regulation of chemerin and CMKLR1 expression in rodent WAT under different physiological conditions in relation to nutritional status, age, gender, pregnancy status, and type of diet. Although studies have revealed a similar degree of expression of chemerin and CMKLR1 in epididymal, perirenal, and mesenteric (visceral) fat depots, it is important to note that in epididymal WAT, the levels of RNA expression between chemerin and CMKLR1 are more similar.

## 2. Results

### 2.1. Influence of Age and Gender on Chemerin and CMKLR1 Protein Expression

**Figure 1:**
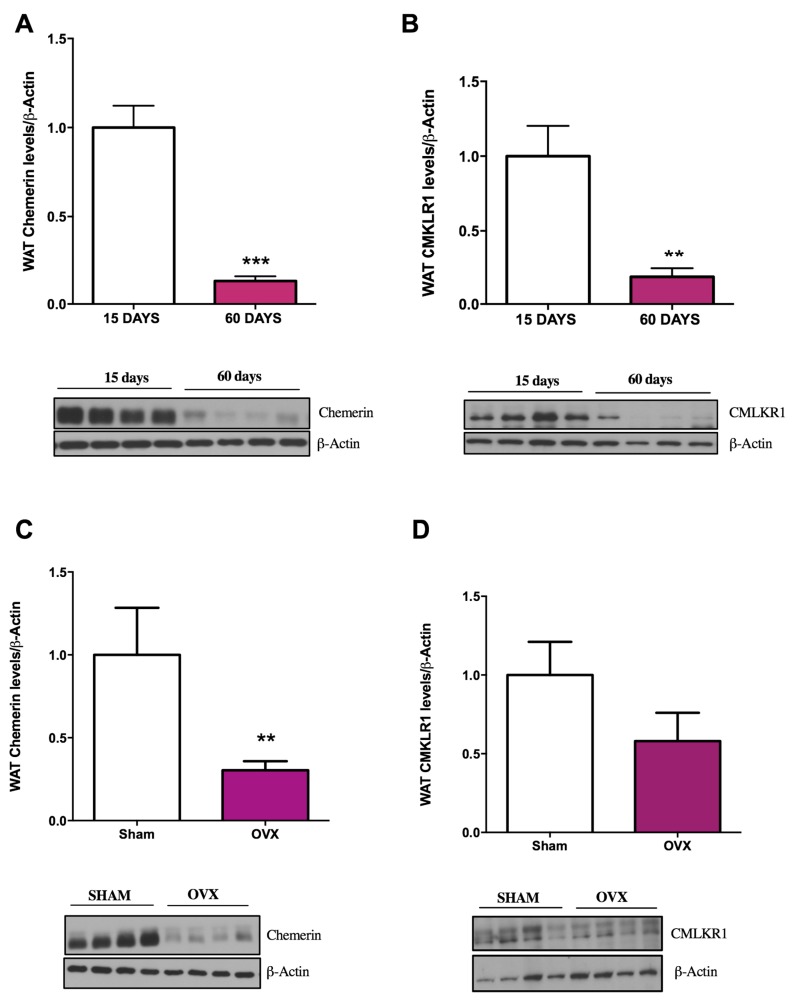
Postnatal development, age, and sex steroids affect chemerin and chemokine-like receptor (CMKLR1) protein expression in gonadal white adipose tissue (gWAT) of female rats. (**A**,**B**) Quantification of the immunoblot data and a representative immunoblot (**lower panel**) of chemerin and CMKLR1 during postnatal development (15 days and 60 days of age) in female rats. (**C**,**D**) Quantification of immunoblot data and a representative immunoblot (**lower panel**) of chemerin and CMKLR1 relative to control (Sham) two weeks after ovariectomy (OVX). Expression of the indicated proteins was normalised to β-actin as the control. Data are expressed as mean ± SEM (*n* = 7 to 8 animals per group); ** *p* < 0.01, *** *p* < 0.001: 15 days vs. 60 days and OVX vs. controls.

**Figure 2:**
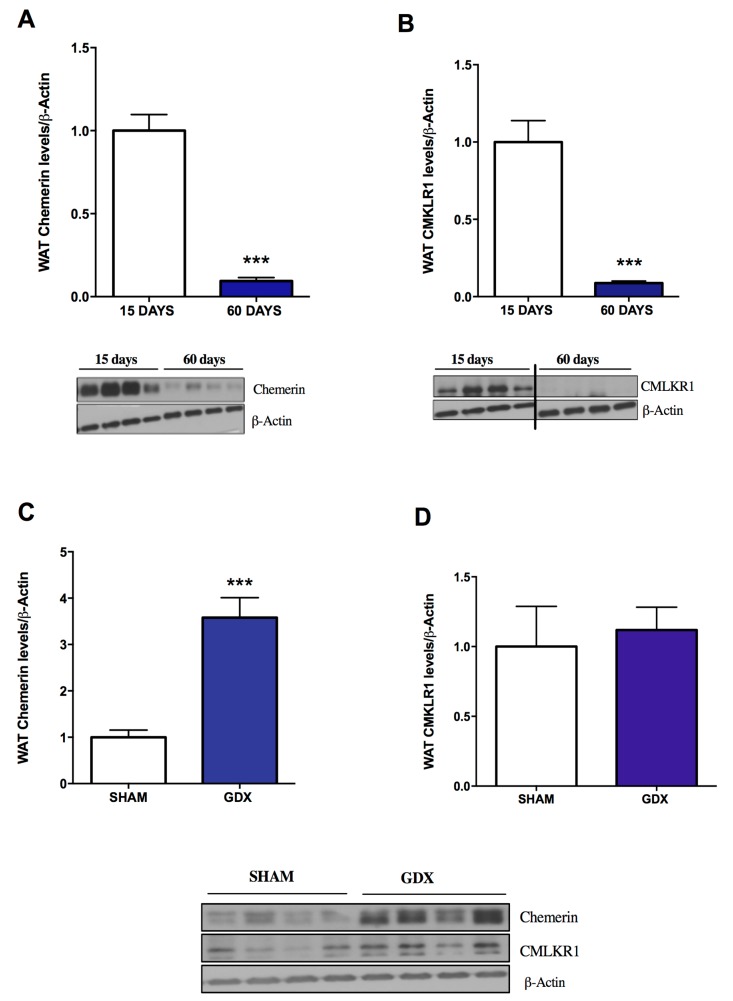
Effects of age and sex steroids on chemerin and CMKLR1 protein expression in gWAT of male rats. (**A**,**B**) Quantification of immunoblot data and a representative immunoblot (**lower panel**) of chemerin and CMKLR1 during postnatal development in male rats (15 days and 60 days of age). (**C**,**D**) Quantification of immunoblot data and a representative immunoblot (lower panel) of chemerin and CMKLR1 relative to control (Sham) two weeks after gonadectomy (GDX). Expression of the indicated proteins was normalised to β-actin as the control. Data are expressed as mean ± SEM (*n* = 7 to 8 animals per group); *** *p* < 0.001: 15 days vs. 60 days and GDX vs. controls.

Firstly, we decided to test whether the expression of both the ligand and the receptor is influenced by postnatal development. Therefore, we assessed their levels in WAT taken from either 15-day-old (pre-pubertal stage) or 60-day-old (post-pubertal stage) rats. Of note, we found marked differences in the expression of both the ligand and receptor with a marked decrease in the expression of both in WAT taken from older animals (60 days) of both genders (Figure 1A,B and Figure 2A,B).

In addition in order to assess the presence of sexual dimorphism, we compared the expression of chemerin and CMKLR1 in both genders (Figure S1B–E). We found higher levels of chemerin in males than in females at the prepubertal stage (15 days).

This difference in the pattern could be due, among other factors, to the marked upregulation of gonadal hormones, which is one of the main features of this stage of development; alternatively, the reason may be the intrinsic process related to postnatal WAT development. To gain some insight into this issue, a similar experiment was carried out on animals of both genders after either ovariectomy or orchidectomy. We found that sham-operated rats had similar expression levels of CMKLR1 in comparison with gonadectomised animals, either after ovariectomy (females) or orchidectomy (males; Figure 1D and Figure 2D). These data implied that the expression of this receptor is not under the influence of the surge of gonadal hormones related to puberty.

On the other hand, the changes in the expression of chemerin showed a completely different picture. We found that its expression was attenuated in ovariectomised female rats (Figure 1C) and increased in orchidectomised male animals (Figure 2C) in comparison with their respective sham-operated controls. These data suggested that at this stage of development, testicle-derived hormones exert an inhibitory action on chemerin expression in WAT, whereas ovary-derived hormones have an opposite effect.

To investigate the correlation between tissue and circulating levels we measured chemerin in serum for both genders in control and orchidectomised rats (Figure S1A). Similar levels were found in all the groups assessed.

### 2.2. Influence of Food Deprivation and Leptin on Chemerin and CMKLR1 Protein Expression

**Figure 3:**
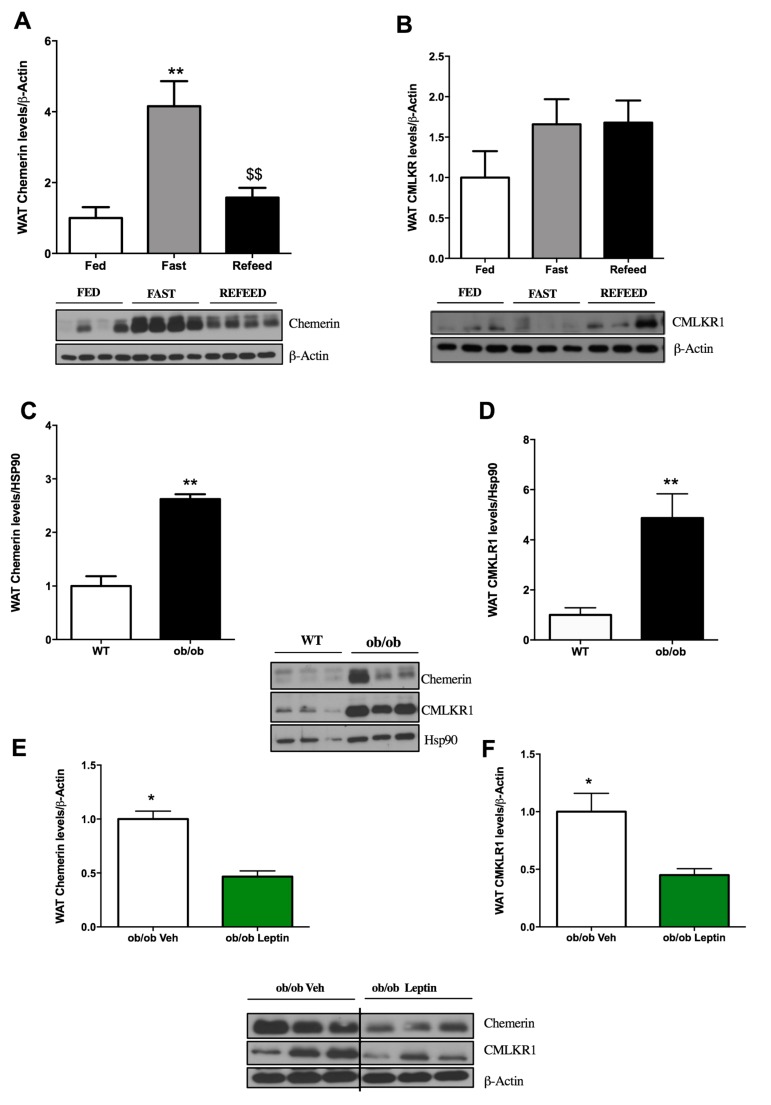
Nutritional status and leptin exert effects on chemerin and CMKLR1 expression in gWAT. (**A**,**B**) Quantification of immunoblot data and a representative immunoblot (**lower panel**) of chemerin and CMKLR1 in gWAT of *ad libitum* fed mice and mice after 24 h fasting and 24 h refeeding. (**C**,**D**) Quantification of immunoblot data and a representative immunoblot (**middle panel**) of chemerin and CMKLR1 in gWAT in wild-type and ob/ob mice. (**E**,**F**) Quantification of immunoblot data and a representative immunoblot (**lower panel**) of chemerin and CMKLR1 in gWAT of ob/ob mice treated with vehicle (veh) or leptin. Expression of the indicated proteins was normalised to β-actin or heat shock protein 90 (HSP90) as the control. Data are expressed as mean ± SEM (*n* = 7 to 8 animals per group); * *p* < 0.05, ** *p* < 0.01: fasting vs. controls; ^$$^ *p* < 0.01: refed vs. controls.

After we determined the influence of gender and gonadal function on the expression of chemerin and its receptor in WAT, we decided to perform further experiments on male rats. We started analysing the effect of nutritional status. After fasting, we found a marked statistically significant increase in the expression of chemerin (Figure 3A) but not of CMKLR1 in WAT (Figure 3B). The effect of fasting on chemerin expression was reversed by short-term refeeding (Figure 3A).

The effect of fasting on chemerin expression could be mediated by a variety of mechanisms including products derived from intermediary metabolism and/or signalling via hormones. Among the latter, the obvious candidate was leptin, an adipocyte-synthesised hormone whose levels markedly decrease during fasting and that is well known to perform a major function in the neuroendocrine and peripheral response observed at the tissue level in response to food deprivation. To gain deeper insight into this issue, firstly, we assessed chemerin expression in the WAT of leptin-deficient animals, namely ob/ob mice. We found that ob/ob mice had much greater levels of expression than did wild-type mice (Figure 3C,D). Thus, these data indicated that in the absence of leptin, there was marked upregulation of both the ligand and receptor, suggesting that physiological leptin levels are important for the regulation of the chemerin system. Further support for this notion came from an experiment showing that ob/ob mice treated with leptin had clearly lower chemerin levels (Figure 3E) and chemerin receptor levels (Figure 3F).

### 2.3. Chemerin and CMKLR1 Gene Expression in Two Hyperleptinemic Experimental Settings: A High-Fat Diet (HFD) and Pregnancy

**Figure 4:**
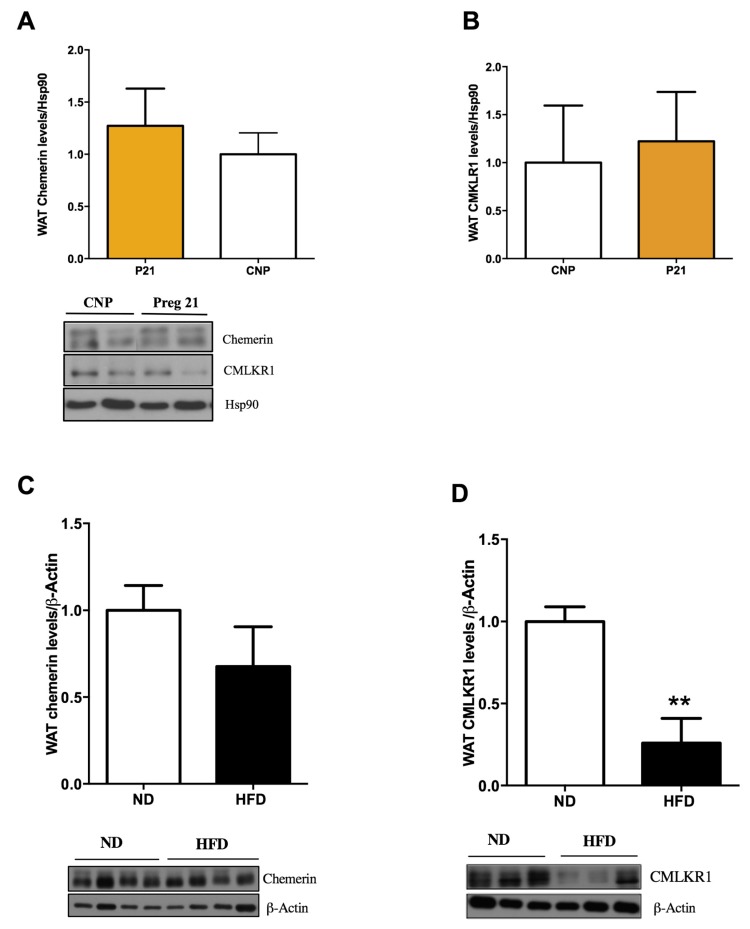
Effects of gestation and a high-fat diet (HFD) on chemerin and CMKLR1 protein expression in gWAT. (**A**,**B**) Quantification of immunoblot data and a representative immunoblot (lower panel) of chemerin and CMKLR1 in control (non-pregnant) rats and at 21 days of gestation. (**C**,**D**) Quantification of immunoblot analysis and a representative immunoblot (**lower panel**) of chemerin and CMKLR1 in rats on the HFD and in the control (Standard diet). Expression of the indicated proteins was normalised to β-actin or HSP90 as the control. Data are expressed as mean ± SEM (*n* = 7 to 8 animals per group); ** *p* < 0.01: HFD vs. controls.

Because of the above-mentioned data regarding the influence of leptin on the chemerin system, we decided to further study this issue by assessing the expression of both chemerin and CMKLR1 in WAT samples collected from animals exposed to either an HFD or pregnancy. Apart from the intrinsic value of data generated with these models, they share a common feature, namely that they involve hyperleptinemia and leptin resistance, at least in terms of the central regulation of energy balance. Our results revealed that chemerin protein levels in WAT were only slightly affected by the HFD (Figure 4C), in contrast to a marked decrease observed in CMKLR1 levels in comparison with the tissues from animals exposed to the standard diet (Figure 4D). These data indicated that the expression of the receptor, but not the ligand, was markedly influenced by the diet. To determine whether this effect is mostly mediated by leptin resistance, we studied a physiological model, namely, pregnancy. Contrary to our expectations, we did not detect any significant differences in chemerin (Figure 4A) or CMKLR1 (Figure 4B) protein expression at 21 days of pregnancy compared with the control (non-pregnant). The discrepancy between these two models of leptin resistance may be likely due to the fact that pregnancy is an experimental physiological model in which many other endocrine and metabolic changes are present.

## 3. Discussion

In this study, we demonstrated that chemerin and CMKLR1 are regulated in the WAT by different conditions associated with metabolic changes such as a stage of postnatal development, gonadal function, food deprivation, and diet. In general, the most interesting finding of our study is the differential regulation of this ligand and receptor in the WAT collected from rodents exposed to the same experimental conditions. These findings are important in light of the diverse roles ascribed to the chemerin system in the context of different biological processes such as inflammation, adipogenesis, angiogenesis, and metabolic or energy homeostasis [11] to name just a few.

Data gleaned in recent years after the identification of this adipokine showed alterations of its expression in various inflammatory and metabolic diseases such as psoriasis [28], obesity [15,29], type 2 diabetes [26,30], and metabolic syndrome [26,30]. However, in contrast to other adipokines, chemerin’s mechanisms of action are likely to be more complex because it may act via autocrine, paracrine, or endocrine mechanisms [31]. In practical terms, the obvious implication is that its biological effects are likely context-dependent, and there are differences in specific-tissue expression. Additionally, in contrast to many other secreted proteins, chemerin’s regulation is quite complex. There is transcriptional regulation at the tissue level, and adipose tissue has emerged as an important tissue because of its expansion in obesity. Chemerin is secreted in an inactive form, after which it is cleaved and processed either in the serum or tissue. This process, poorly characterised, leads to the formation of different chemerin isoforms that may have different bioactivities and functions

Because the chemerin system is considered quite relevant in terms of adipocyte biology and is a potential therapeutic target in obesity, we focused our study on assessing the expression of this system in WAT.

In terms of adipocyte function and adipokine production, one of the most interesting biological processes is adipogenesis [16]. The strong link between adipokines and gonadal function—in particular at the onset of puberty—is well established [7,32]. Thus, it is known that the onset of puberty is much more influenced by adiposity than by chronological age [33]. Among the different adipokines known to participate in the process of puberty onset, the involvement of leptin is well described. On the other hand, gonadal hormones also markedly influence the production of leptin and other adipokines by adipocytes [32]. In light of this observation, and because chemerin has been shown previously to be expressed in gonadal [10,15,16,34] or placental tissues [10,35], we decided to study its expression in WAT of prepubertal and post-pubertal rodents. Our data show a gender-independent decrease in the expression of chemerin and its receptor at post-pubertal stages in comparison with pre-puberty. To find out whether these changes are related to gonadal function, we performed additional experiments on gonadectomised animals. We observed divergence in the regulation of the expression of chemerin and its receptor. The expression of CMKLR1 was found to be independent of gonadal function. In contrast, the expression of the ligand was influenced in opposite ways by ovarian and testicular functions. Whether the effects of gonadal function on chemerin expression are mediated by sex steroids or other hormones is yet to be studied

The lack of correlation between tissue levels and circulating chemerin levels in relation to gonadal function may look puzzling at first glance. However, it should be noted that chemerin is secreted as an 18-kDa inactive proprotein, known as prochemerin, that can be rapidly converted into its active 16-kDa form by the proteolytic removal of the C-terminal amino acids. This is important because it was found that total serum chemerin protein does not consistently correspond with chemerin bioactivity [36,37] and that the commercial enzyme-linked immunosorbent assay (ELISA) detects the active and the inactive forms of chemerin.

The primary function of adipose tissue is energy storage, but the secretion of different adipokines contributes to the regulation of energy and metabolic homeostasis by controlling glucose uptake, lipolysis, and food intake in rodents and humans [4,5]. There is a wealth of experimental data showing that a loss of chemerin or CMKLR1 abrogates adipocyte differentiation and modifies the expression of genes crucial for glucose and lipid metabolism [12,38,39]. In addition, chemerin has been proposed as a link between obesity and type 2 diabetes. Data obtained in humans have revealed alterations in chemerin levels in different states such as inflammatory diseases (psoriasis, arthritis, inflammatory bowel disease) or in patients with obesity, type 2 diabetes, and/or metabolic syndrome compared with lean and healthy subjects [20,30,40,41], hence, the relevance of gaining further insight on our understanding of the mechanisms regulating adipose tissue levels of both the ligand and the receptor in relation to food intake and adiposity.

Although there are other reports assessing the influence of partial food-deprivation and long term-fasting on chemerin expression in WAT depots, we decided to study the expression of both the ligand and the receptor because studies addressing the latter are scarce. Thus, we evaluated the influence of short-term fasting (24 h). We found a clear increase of protein expression for both chemerin and CMKLR1 that were reversed following refeeding. Since fasting-associated responses in the adipocyte function are leptin-mediated, the refeed decreased the leptin levels to almost basal levels. As leptin decreases in fasting states we tested whether this effect was mediated by leptin. Our data, which shows an increase in both chemerin and CMKLR1 levels in leptin-deficient animals indicates the relevance of leptin to the expression of both proteins. In addition, the results with exogenous leptin administration confirmed that this was indeed a leptin-dependent mechanism. Because ob/ob mice are a standard model for the study of leptin effects or of monogenic obesity, other research groups have published different results on the measurement of the mRNA expression, where chemerin expression in wild-type and ob/ob mice is similar, but these mutant mice show an increase in the expression of CMKLR1; these findings indicate divergence in the regulation of the expression of chemerin and its receptor [36].

Our data on the expression of the ligand in WAT, at least at first glance, contradict some other studies. This discrepancy may be due to differences in the experimental design related to the duration of fasting or in some instances to the use of a different animal model such as chronic and partial food deprivation [42]. In addition, some differences can be attributed to the fact that our data are based on western blot protein measurements, whereas others look at mRNA levels [42]. Whatever the reason for these discrepancies, we found that chemerin protein levels in WAT were similar in tissue samples taken from control and HFD-fed animals, but there was a decrease in CMKLR1 expression in the latter. The finding that the receptor is downregulated in the context of normal chemerin levels indicates that HFD possibly leads to a chemerin-resistant state like the one described for other adipokines such as leptin. On the other hand, the lack of changes in the expression of either the ligand or receptor at the end of gestation indicates that their expression levels in WAT are unlikely to mediate the profound metabolic changes observed at the end of pregnancy, and that the leptin-resistant state of pregnancy is overcome by other endocrine and/or metabolic changes associated with it.

In summary, over the last few years, there has been a steady increase in published data on the regulation of chemerin, an adipokine for which WAT is the main site of synthesis. Our data provide new insights into the regulation of this novel adipokine and its receptor CMKLR1 in WAT. In particular, we obtained data regarding the chemerin and CMKLR1 protein expression in WAT depots collected from animals exposed to different experimental paradigms. These experiments allowed us to document the influence of pubertal development, gonadal function, diet, and leptin signalling on their expression. The integration of this basic knowledge (in the proper context) with the extensive clinical data, which are currently being generated, should lead to a better understanding of the pathophysiological role of chemerin and its potential as a therapeutic target.

## 4. Materials and Methods

### 4.1. Animal Procedures

Male and female Sprague Dawley rats that were bred in the Animalario Xeral of USC (Santiago de Compostela, Spain); obese leptin-deficient ob/ob and C57BL/6 mice (Charles River, Barcelona, Spain), were housed under conditions of controlled temperature (23 °C), illumination (12-h light/dark cycle) and water *ad libitum*. Depending on the experimental setting, animals were fed with chow or high-fat diet, *ad libitum*, or fasted. We used 8 animals per group in each experiment.

We use animals of both sex in the experiments related to sexual dimorphism. All the other studies were carried out in male animals because their absence of oestrous cycle facilitates the interpretation of data obtained and to reduce as much as possible the number of animals used.

After the experiments, the animals were euthanised, and gWAT was rapidly extracted and immediately frozen on dry ice; we kept these samples at −80 °C until analysis.

All the animal procedures were conducted in accordance with the standards approved by the Faculty Animal Committee at the University of Santiago de Compostela, and the experiments were performed in compliance with the Rules of Laboratory Animal Care and International Law on Animal Experimentation and permission number 15010/17/002 (19 February 2018).

#### 4.1.1. Influence of the HFD on Chemerin and CMKLR1 Expression in WAT

In this experiment, 3-week-old male Sprague Dawley rats were randomly assigned standard chow or HFD (Research Diets D12,492; 60% fat, 5.24 kcal g^−1^, Research Diets, New Brunswick, NJ, USA) for 12 weeks and then were euthanised.

#### 4.1.2. Influence of Food Deprivation and Leptin in Chemerin and CMKLR1 Expression in WAT

Adult male Sprague Dawley rats were randomly assigned to 3 treatment groups: Rats with free access to chow diet, rats fasted 24 h and rats fasted 24 h and then refed 24 h.

For testing how leptin affects chemerin and CMKLR1 protein expression in gonadal white adipose tissue, we used C57BL/6J mice as control versus adult male ob/ob leptin-deficient mice at 10 weeks old. For leptin treatment, leptin-deficient mice were divided into 2 groups: ob/ob mice treated with vehicle, and ob/ob mice intraperitoneally injected with human recombinant leptin (0.5 µg/g) provided by Dr A. F. Parlow (National Hormone and Peptide Program, Harbor-UCLA Medical Center, Torrance, CA, USA) every 12 h over 3 days [43].

#### 4.1.3. Influence of Age and Gender on Chemerin and CMKLR1 Expression in WAT

We used male and female Sprague Dawley rats of 15 and 60 days postnatal. Rats were fed *ad libitum* with a standard diet and free water access. After keeping housed one week, they proceeded to be sacrificed on the day they reached the respective age (days 15 and 60).

To test whether there are influences of gonadal hormones, adult male and female Sprague Dawley rats were bilaterally orchidectomised (ORX) or ovariectomised (OVX). Another group was added: Sham, where we performed a control operation as previously described [44,45]. The rats were fed *ad libitum* with a standard diet with free water access for 15 days and then were euthanised.

#### 4.1.4. Influence of Pregnancy in Chemerin and CMKLR1 Expression in WAT

It is well known that pregnancy is a physiological model characterised by increased food intake, fat mass, and hyperleptinemia. To determine whether there is any impact of gestation on chemerin and CMKLR1 expression in WAT, we set up two groups: Rats pregnant for 21 days and nonpregnant rats as controls as described previously [46].

### 4.2. Analysis of WAT Protein Expression by Western Blotting

Total-protein samples were extracted from gWAT, and western blots were performed as previously described [47,48]. Briefly, total-protein samples prepared from lysates from each tissue (20 µg) were subjected to sodium dodecyl sulfate–polyacrylamide gel electrophoresis (SDS-PAGE), electro-transferred onto an Immun-Blot^®^ polyvinylidene difluoride membrane (Bio-Rad, Hercules, CA, USA), and probed with antibodies against chemerin and CMKLR1. Dilutions of primary antibodies were 1:1000, and we employed mouse anti-chemerin (ALX-804-868-c150, Enzo Life Sciences, Farmingdale, NY, USA), rabbit anti-CMKLR1 (ab64881, Abcam, Cambridge, UK), and mouse anti-β-actin (A5316, Sigma Aldrich, St. Louis, MO, USA) antibodies. Secondary antibodies were purchased from Dako and used at dilution 1:5000 in 3% BSA in TBS-T (Tris-buffered saline with 0.1% Tween 20). For protein detection, we used horseradish peroxidase-conjugated secondary antibodies (Dako, Glostrup, Denmark) and chemiluminescence (Pierce ECL Western Blotting Substrate, Thermo Scientific, Waltham, MA, USA). Membranes were then exposed to X-ray films (Super RX, Fuji Medical X-Ray Film, Fujifilm, Minato, Japan) and developed with developer and fixing liquids (AGFA, Mortsel, Belgium) under appropriate dark-room conditions.

Autoradiographs were scanned, and the band signals were quantified by densitometry using the ImageJ 1.33 software (NIH, Bethesda, MD, USA). Representative images for all proteins are shown; each protein’s data were normalised to its own internal β-actin control or HSP90.

### 4.3. Analysis of Levels of Circulating Chemerin

The quantitative determination of chemerin concentrations in serum was conducted by an ELISA in males, female controls, and orchidectomised rodents by means of reagent kits and methods provided by LifeSpan Biosciences, Inc. (Seattle, WA, USA)

### 4.4. Statistical Analysis

The results are expressed as mean ± SEM. GraphPad Prism (5.03) was employed for data analysis. Values are plotted as the mean ± SEM for each genotype. Statistical analysis was performed by one-way analysis of variance ANOVA followed by a post hoc multiple-comparison test (Bonferroni’s test). Data with a p value less than 0.05 were considered statistically significant.

## Supplementary Materials

Supplementary materials can be found at http://www.mdpi.com/1422-0067/19/10/2905/s1.
