# Supplementary material for: Regulation of Chemerin and CMKLR1 Expression by Nutritional Status, Postnatal Development, and Gender
Source: Int J Mol Sci. 2018 Sep 25;19(10):2905. doi: 10.3390/ijms19102905 (PMC6213800; doi:10.3390/ijms19102905)
Supplement: Supplementary file 1 [file ijms-19-02905-s001.pdf]

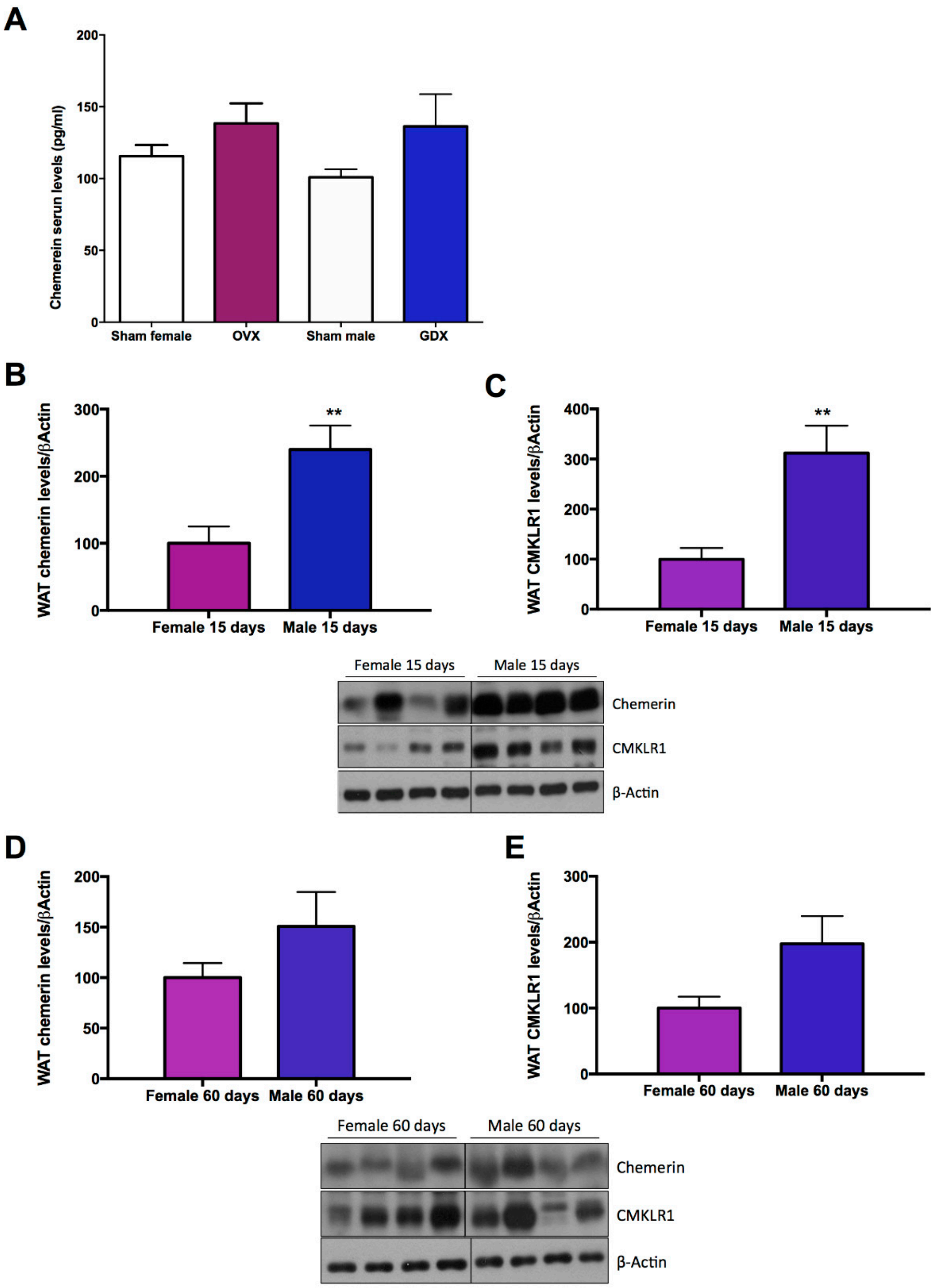

Figure S1. (**A**) Circulating chemerin levels measure by ELISA in males and females control (Sham) and two weeks after orchidectomy (OVX, GDX). (**B,C**) Quantification of immunoblot analysis and representative immunoblot (lower panel) of chemerin and CMKLR1 at 15 days old in female and male rats. (**D,E**) Quantification of immunoblot analysis and representative immunoblot (lower panel) of chemerin and CMKLR1 at 60 days old in female and male rats. Expression of indicated proteins was normalized to β-actin to the control. Data are expressed as mean ± SEM (n = 7–8 animals per group). **$p < 0.01$; male vs. female.
